# Meta-Analysis to Determine Normative Values for the Special Judo Fitness Test in Male Athletes: 20+ Years of Sport-Specific Data and the Lasting Legacy of Stanisław Sterkowicz

**DOI:** 10.3390/sports7080194

**Published:** 2019-08-16

**Authors:** Katarzyna Sterkowicz-Przybycień, David H. Fukuda, Emerson Franchini

**Affiliations:** 1 Department of Gymnastics and Dance, Institute of Sport Sciences, University of Physical Education, 31-571 Krakow, Poland; 2 School of Kinesiology and Physical Therapy, Institute of Exercise Physiology and Rehabilitation Science, University of Central Florida, Orlando, FL 32816, USA; 3 Martial Arts and Combat Sports Research Group, Sport Department, School of Physical Education and Sport, University of Sao Paulo, Sao Paulo 05508-030, Brazil

**Keywords:** martial arts, systematic review, athletic performance, age groups

## Abstract

The aim of this study was to evaluate Special Judo Fitness Test (SJFT) results specific to the population of male judoka and to develop age category norms for junior and senior athletes. A systematic review of the existing literature was conducted to identify 281 publications reporting SJFT results between 1995 and 2018. The final meta-analysis included data from 37 relevant studies that reported SJFT results from 51 individual samples of 515 senior and 209 junior male athletes. The combined mean and SD for SJFT variables were calculated, and the Cohen’s d effect size with 95% confidence intervals (CI) for the senior and junior age classifications were compared. Senior athletes demonstrated higher total number of throws (*d* = 0.41, CI = 0.25–0.57, *p* <0.001) and heart rate (HR) immediately after the SJFT (*d* = 0.18, CI = 0.02–0.35, *p* = 0.025) with limited differences for HR one minute after the SJFT between groups. The SJFT index was lower for seniors compared to juniors (*d* = 0.38, CI = 0.22–0.54, *p* <0.001) indicating better overall performance by the more advanced athletes. Percentile rankings were used to develop SJFT classificatory tables for male senior and junior judo athletes. Training staff can use the age group classifications in the evaluation process of their athletes and for the purpose of monitoring training.

## 1. Introduction

The assessment and evaluation of athletes has evolved to include comprehensive monitoring programs consisting of both general and sport-specific testing. In an effort to simulate the high-intensity intermittent nature of the Olympic sport of judo, a variety of sport-specific tests have been developed that mimic both the technical-tactical actions and energy systems utilized during competition or training [1,2,3,4]. Originally devised by the late Stanisław Sterkowicz in 1995, the Special Judo Fitness Test (SJFT) is now the most popular and well-researched of these assessments [5]. The SJFT meets the desired criteria of objectivity, reliability and validity [6], as confirmed by a large number publications examining the physical preparation of judo athletes [1,7,8,9]. Furthermore, strong correlations support the sensitivity and potential usefulness of this tool in the areas of physiology [7,10], biomechanics [11], and psychology [12]. The ecological utility of the SJFT, including the ability to perform within the training environment, rather than in a laboratory setting, and its relative simplicity represent an efficacious methodology for the periodic monitoring and sport-specific evaluation of judo athletes. In 2010, the SJFT was recommended by the Polish Ministry of Sport and Tourism for regular assessment of athletes in clubs as well as the national team [13].

The SJFT is divided into three periods (A = 15 s; B and C = 30 s) separated by 10-s intervals of passive rest. During each period, the athlete being evaluated sprints between and throws each of the two partners of similar height and body mass using the one-armed shoulder throw (ippon-seoi-nage) as many times as possible. The athlete begins the test placed 3 m from each partner. Immediately following and one minute after the test, heart rate is measured. Stopwatches and heart rate monitors can be employed to aid in the testing procedures [14]. The SJFT index is calculated as follows:

(1) $$SJFT\;Index=\frac{Final\;HR\;\left(bpm\right)+HR\;1\min\;\left(bpm\right)}{Throws\;\left(N\right)}$$

where Final HR is the heart rate recorded immediately after the test; HR 1 min is the heart rate obtained 1 min after test; and Throws is the number of throws completed during the test. Within this context, lower SJFT Index values are indicative of superior fitness and sport-specific performance. Visualization of the Special Judo Fitness Test is available on the Internet [15].

With this unique approach, the individual variables and resulting index from the SJFT provide insight into several key judo-specific factors, including maximal performance, technical execution, fatigability, and recovery. This insight coupled with the possibility of detecting even small changes in fitness during preparation for competition [16] supports the popularity of SJFT across various demographic characteristics. The SJFT has been used in the examination of numerous situations related to performance, including the effects of rapid weight loss [17], recovery type between matches [9], pre-exercise warm-up strategies [18], long-term training programs [19,20,21,22,23], and laterality [24]. With respect to dietary supplementation, scientific studies have evaluated the effects of creatine malate [25], sodium bicarbonate [26], creatine monohydrate [27], and caffeine ingestion [28] on SJFT performance. A more comprehensive review of the available literature on the topic of the SJFT is presented in the recent book chapter by Sterkowicz-Przybycień et al. [29].

In the absence of available data, the provisional test norms developed for men practicing judo [14] have been used in the evaluation of athletes regardless of sex or age category. Due to the potential miscategorization of women, our original meta-analysis focused on developing normative data for the SJFT in this population [30]. Using this methodology, we synthesized the previously reported findings on an international scale, thereby indicating samples deviating from median values, and established norms for junior and senior women. However, to date, comparable data do not exist for male judoka relative to age categories, and reports on differences in SJFT performance between younger and older athletes are ambiguous [31,32]. Therefore, the main aim of this study was to evaluate the SJFT results specific to the population of professional judoka and to develop age category norms for junior and senior athletes.

## 2. Materials and Methods

A systematic review and meta-analysis of the available literature reporting results from the SJFT was conducted following Preferred Reporting Items for Systematic Reviews and Meta-Analysis [33]. The keywords “judo”, “fitness”, “special”, and “SJFT” were utilized to search relevant publications in the following international databases: SportDiscus with full text, MEDLINE, Web of Science, Academic Search Complete, EBSCOhost, and Google Scholar, and papers cited in the original retrieved investigations were searched manually. The inclusion criteria were: (1) data on the age of study participants; (2) competitive level; (3) reporting of sample size and means and standard deviations (SD) for all four SJFT variables; and (4) male participants. The exclusion criteria were: (1) omission of age data; (2) omission of means and standard deviations for SJFT data; and (3) female participants. Publications from January 1995 through December 2018 were included. After identification in databases, the studies were assessed through title and abstracts. Duplicates and studies that did not meet inclusion criteria were excluded. The languages of studies were English, Polish, Russian, and Portuguese, and the data concerned samples of athletes ranging from novice to those at the national or international competitive level. All included study outcomes were complete and not selectively reported. Age categories were determined according to the International Judo Federation senior (>21 years old) and junior (≤21 years old) age classifications [34].

The combined means and SD for number of throws completed during the test, the heart rate values immediately after and 1 min after the test, and the SJFT index were calculated according to equations recommended by Kirkendall et al. [6].

(2) $$Combined\;\bar{x}=\frac{\sum^{\;}\left({\bar{x}}_{i}\cdot \;n_{i}\right)}{\sum^{\;}n_{i}}$$

(3) $$Combined\;SD=\sqrt{\frac{\sum^{\;}\left(n_{i}\;\cdot \;SD_{i}^{2}\right)}{\sum^{\;}n_{i}}}$$

where ${\bar{x}}_{i}$ is the mean value for a given sample, $n_{i}$ is the sample size for a given sample, and $SD_{i}$ is the standard deviation for a given sample. Subsequently, the Cohen’s *d* effect size with 95% confidence intervals for the senior and junior age classifications for each of the parameters of SJFT using combined $\bar{x}$ and combined SD was calculated. The following interpretation of Cohen’s *d* effect sizes were employed: small = 0.20, medium = 0.5, large = 0.80 [35]. A random effects meta-analysis was utilized to evaluate the relevant samples for the SJFT Index. To indicate the extent of heterogeneity between individual samples the Cochran *Q* test and *I*^2^ statistic (range from 0% to 100%) were computed. The interpretation of *I*^2^ values was completed as follows: low ≤25%, moderate 20–50%, and high ≥75%.

Percentile rankings considering a 5° scale were used to develop SJFT classificatory tables for male senior and junior judo athletes in the following categories: Excellent = highest 5% or above the 95th percentile, Good = next highest 15% or between the 80th and 95th percentiles, Regular = middle 60% or between the 20th and 80th percentiles, Poor = next lowest 15% or between the 5th and 20th percentiles, and Very poor = lowest 5% or below the 5th percentile. The statistical calculations were made using the ‘metafor’ package [36] running under R Environment [37], SPSS 21, and Excel.

## 3. Results

**Figure 1:**
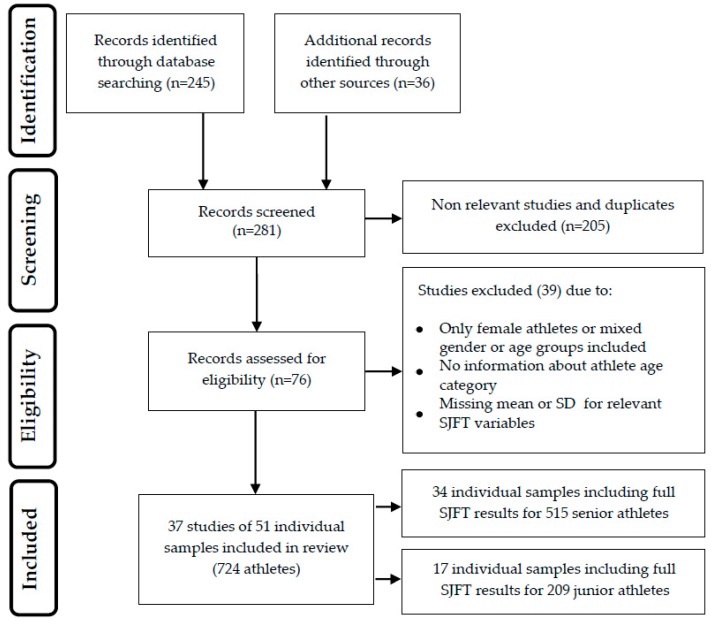
Flow diagram of the literature search procedure. SJFT = Special Judo Fitness Test.

The results of the different phases of the systematic literature search are illustrated in Figure 1. A total of 281 documents were initially identified. During the screening stage, duplicates and ineligible papers were removed. At the eligibility stage, 39 studies that did not meet the inclusion criteria were excluded. The final analysis included data from 37 relevant studies that reported SJFT results from 51 individual samples of 515 senior (Table 1) and 209 junior male judo athletes (Table 2).

**Figure 2:**
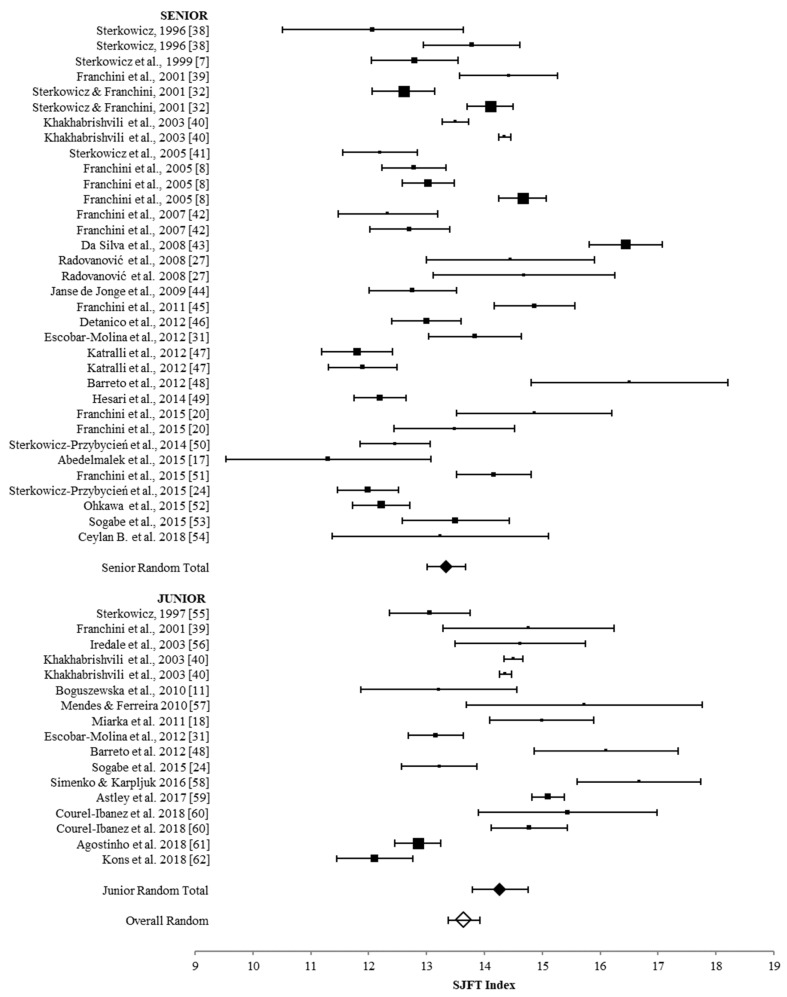
Forest plot summarizing the meta-analysis for Special Judo Fitness Test (SJFT) Index with mean values and 95% confidence intervals.

The results of the meta-analysis for SJFT Index in the senior (*n* = 515) and junior (*n* = 209) judo athletes are illustrated in Figure 2. High heterogeneity was found for seniors (*Q* = 579.9, *p* <0.001, *I*^2^ = 94.3), juniors (*Q* = 216.0, *p* <0.001, *I*^2^ = 92.6), and the combined subgroups (*Q* = 90.3, *p* <0.001, *I*^2^ = 94.4).

Medium effect sizes were identified for the age category comparisons in the SJFT index (Cohen’s *d* = 0.38, 95% confidence interval (CI = 0.22 to 0.54, *p* < 0.001) and number of throws completed (Cohen’s *d* = 0.41, 95% confidence interval (CI = 0.25 to 0.57, *p* < 0.001). A small effect size was identified for HR directly after the test (Cohen’s *d* = 0.18, 95% confidence interval (CI = 0.02 to 0.35, *p* = 0.025), while no effect was shown for HR 1 min after the test (Cohen’s *d* = 0.001, 95% confidence interval (CI = −016 to 0.16, *p* = 0.994) between the combined samples of senior and junior judo athletes. Data collected during the systematic literature review and meta-analysis allowed for the establishment of normative values for all of the SJFT variables. The classificatory data for senior and junior male judo athletes are presented in Table 3.

## 4. Discussion

The main goal of the present systematic review and meta-analysis was to develop a SJFT classificatory table for junior and senior male judo athletes. With this data, judo coaches and strength and conditioning professionals can use the age group classifications in the evaluation process of their athletes. The norms were different between age groups, with one throw more for seniors compared to juniors for all classifications. For HR immediately after the SJFT, lower values need to be achieved by junior judo athletes in order to be classified as Excellent; quite similar values are required for the Good classification, whereas higher values (i.e., inferior cardiovascular responses) characterize the Very poor, Poor and Regular classifications of junior compared to senior judo athletes. For HR 1-min after the SJFT, lower values should be achieved by junior judo athletes in order to be classified as Excellent and Very good, whereas a slower HR recovery characterizes the Very poor, Poor, and Regular classifications of junior compared to senior judo athletes. However, for the SJFT Index, senior judo athletes classifications are marked by lower values (i.e., better results) for senior compared to junior judo athletes. Moreover, it is important to emphasize that the differences between age groups reached medium effect sizes for the total number of throws and SJFT Index and a small effect size for the HR immediately after the test, whereas no effect was found for the HR 1 min after the SJFT.

Previous studies proposed SJFT classificatory tables for senior [14] and junior [61] judo athletes. The first investigation [14] used quintiles to divide the grades and had a broad range of judo athletes as subjects (141 athletes, from 16 to 34 years of age, with 51 up to 151.5 kg of body mass, 159 to 200 cm of height, and varying from 3rd kyu to 3rd dan). However, the classification established in that study resulted in lower limits for total number of throws in the Excellent and Good classifications; a similar value was found for the Regular classification, whereas the Poor and Very poor classifications had higher limits for the seniors in the present study. For juniors, similar limits were found in the present study compared to the table proposed by Franchini et al. [14].

For HR immediately after and one minute after the SJFT, our meta-analysis indicated that lower values characterized the Excellent and Good classifications for junior and senior judo athletes compared to the general table [14], whereas an overlap existed for the Regular and Poor classifications, and similar (for seniors) or slightly higher values (for juniors) were found for the Very poor classification. For the SJFT Index, lower values characterized the Excellent, Good, and Regular grades for seniors and the Excellent grade for juniors, whereas all other grades presented an overlap with the table proposed by Franchini et al. [14]. Taken together, these differences indicate that the present table resulted in the need for better performance in order to be classified as Excellent, whereas the reverse is true for the lower classification grades. This is likely due to the difference in percentile references used to develop each table with the current data being based on 5th percentile gradations at the extremes which better represent the best and worst SJFT performances.

Agostinho et al. [61] recently proposed a SJFT classificatory table for high-level junior judo athletes, using the same grade divisions used in the present study. In comparison, the newly developed table exhibits lower limit values for the Excellent, Good, Poor, and Very poor grades in total number of throws; higher values for the Excellent grade in HR immediately after the SJFT; higher values for the Excellent grade and lower values for the Very poor grade in HR one minute after the SJFT; and higher values for the Excellent, Poor, and Very poor grades in the SJFT Index. It is likely that these differences are due to the higher level and homogeneity of the judo athletes (i.e., National Team members) included in the Agostinho et al. [61] study compared to the heterogeneity of samples used in our meta-analysis.

A previous meta-analysis with female judo athletes also identified significantly lower SJFT Index values for seniors compared to juniors [30], although a larger difference between age groups was observed in the study with female athletes compared to the findings of the present investigation. The better performance for seniors compared to juniors can be explained by both greater training ages and the rigors of the national team selection processes. As maximal HR is inversely related with chronological age [63], higher values would be expected for juniors compared to seniors which is confirmed by the lower SJFT classification grades. HR recovery is influenced by several factors, but aerobic fitness seems to be a major contributor (i.e., aerobically-trained individuals present a faster HR recovery) [63]. However, no effect was found for HR one minute after the SJFT, suggesting that junior and senior judo athletes seem to present a similar aerobic fitness. Simultaneously, the SJFT index is correlated with parameters of both anaerobic (30-s Wingate test) and aerobic (graded exercise tests on treadmill) power and capacity, demonstrating the utility of this ecologically valid test that can be used to evaluate the effort tolerance in judo athletes when laboratory tests are not available [7]. Ceylan et al. [54] confirmed large positive correlations between SJFT results and the Wingate test in a group from the Turkish Olympic judo team along with large negative correlations with body fat percentage.

The SJFT is predominantly anaerobic in nature [45], and there is evidence that the total number of throws is related to the anaerobic fitness due to power generation necessary to execute judo throws as well as the required sprints between throws [29]. Consequently, any improvement in the total number of throws is related to an increase in at least a 6-m run (i.e., a minimum of 0.08 m/s improvement in speed) and one throw during the test. Considering that the total duration of effort during the SJFT is 75 s, and that during the test, athletes need to accelerate, decelerate, throw the opponent while changing direction, and accelerate again, an improvement in the total number of throws may be the result of increased speed, muscle power, and agility, either isolated or in combination. Furthermore, the number of throws completed during the SJFT has been shown to be related to technical effectiveness during judo competition, with differences depending on the length of the match and between men and women [62,64]. On the other hand, a lower HR after the SJFT or a faster HR recovery has been interpreted as an improvement in aerobic fitness [29]. Calculated from both of these sets of variables, the SJFT Index may be important for judo performance as many actions require use of the ATP-PCr, glycolytic, and oxidative energy systems [65].

While judo competitions are typically organized by weight category, the data identified during the systematic review process did not allow for the proper development of norms with respect to body mass. These limitations included the lack of reporting such details, the relative heterogeneity of the body masses represented within the literature (though sample data may be assumed to track with population data), and the potential for low sample sizes by weight category, particularly within the lightest and heaviest athletes. Future investigations may focus on expanding upon the current work by accumulating large-scale SJFT data from athletes in different weight categories.

## 5. Conclusions

The present study provides a comprehensive set of normative values for variables from the SJFT for junior and senior male judo athletes. Additionally, an age-group comparison was conducted with senior athletes, demonstrating a higher total number of throws (medium effect) and HR immediately after the SJFT (small effect), with limited differences observed for HR one minute after the SJFT between groups. Moreover, the SJFT index was lower for seniors compared to juniors (medium effect) indicating better overall performance during the SJFT by the more advanced athletes.

This work is dedicated to Professor Stanisław Sterkowicz, creative scientist and originator of the SJFT, who shared his passion for sport and served as a beacon for others. A man with an independent character, who sought innovative solutions and scientific investigations aimed at improving the practice of sport and coaching. He demonstrated how to connect the problems of theory, biomechanics, physiology, and psychology in the context of combat sports in an interdisciplinary manner. He maintained a collaboration with researchers from different countries and backgrounds for decades. He was the owner of black belts in judo, ju-jitsu, karate, and hapkido. Martial arts were his way of life.

## Figures and Tables

**Table 1:** Special Judo Fitness Test (SJFT) results reported in 34 international samples of senior male judo athletes.

						Throws		Final HR (b·min^−1^)		HR 1 min (b·min^−1^)		SJFT Index	
Source	Study	Country	Age Category	Level	*n*	$\bar{x}$	SD	$\bar{x}$	SD	$\bar{x}$	SD	$\bar{x}$	SD
Sterkowicz, 1996 [38]	CS	Poland	Senior	E	10	27.4	4.7	177	9.5	130	7	11.57	2.52
Sterkowicz, 1996 [38]	CS	Poland	Senior	N	10	24	2	182	6.4	136	4.2	13.28	1.34
Sterkowicz et al. 1999 [7]	CS	Poland	Senior	E + N	15	27.3	2.7	181.6	6.2	150	11.8	12.29	1.48
Franchini et al. 2001 [39]	CS	Brazil	Senior	N	6	24.2	1.2	179	11.4	157	15.4	13.92	1.06
Sterkowicz and Franchini 2001 [32]	CS	Brazil and Poland	Senior	E	33	27.7	2.9	180	10	152	18.1	12.1	1.6
Sterkowicz and Franchini 2001 [32]	CS	Brazil and Poland	Senior	N	47	25.1	2.2	185	9.6	156	15.5	13.6	1.4
Khakhabrishvili et al. 2003 [40]	CS	Georgia	Senior	E	7	26.1	0.3	181	0.4	156	0.4	13	0.31
Khakhabrishvili et al. 2003 [40]	CS	Poland	Senior	E	7	24.7	0.3	183	0.4	160	0.6	13.85	0.14
Sterkowicz et al. 2005 [41]	CS	Germany	Senior	E	9	29.1	2.2	183	5.1	156	8.3	11.7	0.99
Franchini et al. 2005 [8]	CS	Brazil	Senior	E	13	28.0	2	179	6	163	10	12.28	1.01
Franchini et al. 2005 [8]	CS	Brazil	Senior	E	23	28	2	181	10	162	12	12.53	1.11
Franchini et al. 2005 [8]	CS	Brazil	Senior	N	53	25	2	186	11	165	13	14.16	1.52
Franchini et al. 2007 [42]	CS	Brazil	Senior	E	7	28	3	178	9	157	11	11.83	1.16
Franchini et al. 2007 [42]	CS	Brazil	Senior	E	13	27	2	175	9	151	7	12.21	1.26
Da Silva et al. 2008 [43]	CS	Brazil	Senior	N	30	20.2	2.7	184	3.7	139	4.2	15.94	1.78
Radovanović et al. 2008 [27]	CS	Serbia	Senior	N	6	24.8	2.8	181	6.4	155	11.8	13.95	1.82
Radovanović et al. 2008 [27]	P1	Serbia	Senior	N	6	24.2	2.5	183	7.8	156	13.1	14.18	1.96
Janse de Jonge et al. 2009 [44]	CS	Australia	Senior	E	12	27.3	2.7	174	10	157	11	12.26	1.34
Franchini et al. 2011 [45]	CS	Poland	Senior	N	14	26	2	196	12	169	9	14.37	1.33
Detanico et al. 2012 [46]	CS	Brazil	Senior	N	18	27	2	179	10	155	15	12.5	1.3
Escobar-Molina et al. 2012 [31]	CS	Spain	Senior	E	10	26	2	183	7	160	15	13.34	1.29
Katralli et al. 2012 [47]	CS	India	Senior	N	20	28.4	2.3	177	9	141	21.6	11.3	1.4
Katralli et al. 2012 [47]	CS	India	Senior	N	11	28.4	2	181	5.7	143	18.2	11.4	1
Barreto et al. 2012 [48]	CS	Brazil	Senior	N	9	21.9	2.9	183.3	12.5	161.3	20.5	16	2.6
Hesari et al. 2014 [49]	CS	Iran	Senior	E + N	19	28	1.8	177	6.8	143	10.5	11.7	1
Franchini et al. 2015 [20]	RM	Brazil	Senior	N	6	25	2	188	10	166	14	14.36	1.68
Franchini et al. 2015 [20]	RM	Brazil	Senior	N	7	25	2	177	13	150	17	12.98	1.4
Sterkowicz-Przybycień et al. 2014 [50]	RM	Poland	Senior	N	7	27.9	1.8	181	10.9	151.7	13.9	11.96	0.82
Abedelmalek et al. 2015 [17]	RM	Tunisia	Senior	N	11	31	2.7	182.3	5.3	154	2.8	10.8	3
Franchini et al. 2015 [51]	RM	Brazil	Senior	N	10	28	1	197	6	178	9	13.66	1.04
Sterkowicz-Przybycień et al. 2015 [24]	CS	Poland	Senior	E + N	17	28	2.4	183	8.2	136	11.9	11.49	1.11
Ohkawa et al. 2015 [52]	CS	Japan	Senior	N	24	27.2	1.9	172	8.6	146	12.8	11.72	1.24
Sogabe et al. 2015 [53]	RM	Japan	Senior	N	18	26.1	3.9	180.6	7	151.7	12.3	13	2
Ceylan et al. 2018 [54]	CS	Turkey	Senior	E	7	27	3	182	10	165	15	12.74	2.52

Throws = number of throws completed during the test; Final HR = heart rate registered immediately after the test; HR 1 min. = heart rate obtained 1 min after the test; CS = cross-sectional study; RM = study with repeated measurements; E = elite; N = non-elite.

**Table 2:** Special Judo Fitness Test (SJFT) results reported in 17 international samples of junior male judo athletes.

						Throws		Final HR (b·min^−1^)		HR 1 min (b·min^−1^)		SJFT Index	
Source	Study	Country	Age Category	Level	*n*	$\bar{x}$	SD	$\bar{x}$	SD	$\bar{x}$	SD	$\bar{x}$	SD
Sterkowicz, 1997 [55]	CS	Poland	Junior	N	11	24.5	1.8	176	14.5	130	20.7	12.55	1.18
Franchini et al. 2001 [39]	CS	Brazil	Junior	N	6	25.3	2.6	193	5.5	167	7.8	14.26	1.85
Iredale et al. 2003 [56]	RM	New Zealand	Junior	N	9	24.3	1.9	187.4	9.4	154	15.4	14.12	1.72
Khakhabrishvili et al. 2003 [40]	CS	Georgia	Junior	E	7	25.4	0.3	188	0.5	162	0.5	14	0.22
Khakhabrishvili et al. 2003 [40]	CS	Poland	Junior	E	7	24	0.3	177	0.4	154	0.4	13.86	0.14
Boguszewska et al. 2010 [11]	CS	Poland	Junior	E	8	25	3.6	187	19.9	129	10.9	12.71	1.94
Mendes and Ferreira 2010 [57]	CS	Brazil	Junior	N	8	22.7	3.4	182	8	155	15.6	15.23	2.94
Miarka et al. 2011 [18]	Control	Brazil	Junior	N	8	23.7	1.4	187	11	154	11	14.49	1.3
Escobar-Molina et al. 2012 [31]	CS	Spain	Junior	E	13	27	2	183	5	153	9	12.66	0.88
Barreto et al. 2012 [48]	CS	Brazil	Junior	N	9	22	2.4	183.4	9.6	156.8	13.9	15.6	1.9
Sogabe et al. 2015 [24]	RM	Japan	Junior	E	9	26.8	2	184.9	7.6	153	10.4	12.72	0.99
Simenko and Karpljuk 2016 [58]	RM	Slovenia	Junior	N	9	22.11	1.83	191.22	6	163.89	12.05	16.17	1.63
Astley et al. 2017 [59]	Control	Brazil	Junior	N	18	23.9	1.7	185.8	9.6	163.4	13.4	14.6	0.6
Courel-Ibanez et al. 2018 [60]	CS	Spain	Junior	N	10	24	3	191	8	159	14	14.94	2.49
Courel-Ibanez et al. 2018 [60]	CS	Spain	Junior	E	12	24	3	187	8	155	11	14.27	1.16
Agostinho et al. 2018 [61]	CS	Brazil, Spain, and Serbia	Junior	E	45	27.4	2.7	180	11	156	17	12.35	1.36
Kons et al. 2018 [62]	CS	Brazil	Junior	N	20	28	3	176	12	143	16	11.6	1.5

Throws = number of throws completed during the test; Final HR = heart rate registered immediately after the test; HR 1 min = heart rate obtained 1 min after the test; CS = cross-sectional study; RM = study with repeated measurements; E = elite; N = non-elite.

**Table 3:** Special Judo Fitness Test (SJFT) classificatory table for male judo athletes.

Classification	Number of Throws		Final HR (b·min^−1^)		HR 1 min (b·min^−1^)		SJFT Index	
	Junior	Senior	Junior	Senior	Junior	Senior	Junior	Senior
Excellent	≥29	≥30	≤165	≤166	≤129	≤130	≤11.04	≤10.47
Good	27–28	28–29	166–173	167–173	130–140	131–141	11.05–12.23	10.48–11.68
Regular	23–26	24–27	174–190	174–188	141–164	142–163	12.24–14.73	11.69–14.22
Poor	21–22	22–23	191–198	189–195	165–175	164–173	14.74–15.92	14.23–15.43
Very poor	≤20	≤21	≥199	≥196	≥176	≥174	≥15.93	≥15.44
